# Impact of COVID-19 on Saudi Children: Special Focus on Behavioral, Social, and Emotional Aspects, 2020-2021

**DOI:** 10.7759/cureus.19856

**Published:** 2021-11-24

**Authors:** Hani S Almugti, Atheer Alotaibi, Ali Almohammed, Rana Abuhadi, Rawdah Baeshen, Ziyad Alharthi, Ahmed Alsharari, Sahar Alotaibi, Yazan Omar, Norah Alturki, Imtenan Oberi, Almunthir Alrehaili, Aghnar Alzahrani, Fatimah Alghanim, Raum Ayoub

**Affiliations:** 1 Epidemiology and Public Health, Ministry of National Guard Health Affairs, King Abdullah International Medical Research Center, King Saud bin Abdul-Aziz University for Health Sciences, Jeddah, SAU; 2 College of Pharmacy, Shaqra University, Shaqra, SAU; 3 College of Medicine, Jordan University of Science and Technology, Irbid, JOR; 4 College of Medicine, Jazan University, Jazan, SAU; 5 College of Medicine, Al-Baha University, Al-Baha, SAU; 6 College of Dentistry, Jouf University, Jouf, SAU; 7 College of Medicine, King Khalid University, Abha, SAU; 8 College of Medicine, King Saud University, Riyadh, SAU; 9 College of Medicine, Batterjee Medical College, Jeddah, SAU; 10 College of Medicine, Imam Abdulrahman Bin Faisal University, Khobar, SAU

**Keywords:** covid-19, social, emotional, behavioral, saudi children

## Abstract

Background

The coronavirus disease 2019 (COVID-19) pandemic has negatively affected many aspects of daily life. In Saudi Arabia, many studies, using a range of assessment approaches, have examined how the pandemic has affected the mental health of both the general public and healthcare workers. However, to develop effective public-health initiatives for such crisis events, it would also be relevant to determine the pandemic’s impact on the behavioral, emotional, and social lives of Saudi children.

Objective

To assess, among Saudi children aged 3-15 years, the behavioral, emotional, and social changes that have occurred in their daily lives due to the COVID-19 pandemic.

Materials and methods

This study featured a cross-sectional design. Potential participants were approached through the most popular social media in Saudi Arabia, and the final sample size was 651 parents. As, at the time of data collection, the members of the Saudi public were requested to avoid face-to-face meetings where possible, a well-designed electronic questionnaire featuring closed-ended questions was used.

Results

Descriptive statistics showed that the mean age of the parents was 29±7 years (range: 20-60 years); over half (58%) were female. During the COVID-19 outbreak, one-third of children had asked to sleep in their parents’ beds. Furthermore, approximately 30% of children demonstrated increased irritability and mood swings when compared with the period before the pandemic. Concerning adaptive social behaviors, during the pandemic, 22% of children appeared calmer and 14% of children seemed more thoughtful. Our study reports more screen time, less physical activity, and reduced sleep time among children compared with the pre-pandemic period.

Conclusion

The COVID-19 pandemic has psychologically affected children. The present results highlight the need to reduce this psychological burden by enhancing children’s emotional resilience and involving parents in health-promotion programs aimed at mitigating the negative impacts of such public-health crises.

## Introduction

Globally, millions of people have been infected with coronavirus disease 2019 (COVID-19), and the trend of infections continues to show volatility [1]. To combat the virus and limit its spread, each country must make important decisions and implement measures to encourage the public to follow practices such as “social distancing” [2]. School closures have been a common public response to the COVID-19 pandemic [3]; thus, in many countries children have spent large amounts of time at home, receiving education through online- or home-based learning approaches.

Several medical studies have reported that, in contrast to adults, children who contract COVID-19 generally experience relatively mild­ illness and lower morbidity [4]. For example, a Saudi-Arabia-based retrospective study of 88 pediatric patients with COVID-19 reported that most of these hospitalized children had experienced relatively mild symptoms, with only a very small number (n=7; 8%) developing severe disease [5]. Several different hypotheses have been proposed to explain children’s relatively positive prognosis and lower susceptibility to COVID-19 [6]. The most reasonable explanatory hypothesis is the lower prevalence of comorbid conditions among children when compared with adults; the risk of critical presentation of COVID-19, as well as death, among pediatric patients, increases with age and among individuals who have other comorbidities, such as heart health problems, cancer, diabetes, and/or pulmonary diseases [6,7].

Besides its health consequences, the COVID-19 pandemic has also affected virtually every other aspect of human life, including social, economic, and political concerns [8]. The social restrictions implemented by governments to control the spread of COVID-19 have caused lifestyle changes and have had a huge impact on people’s mental and physical health [7,8]. Studies have indicated that, when children are not in school, they are less physically active, have more abnormal sleep patterns, have less healthy diets, spend more time viewing digital screens, and generally tend to be more sedentary. All of these are associated with weight gain and declining cardiorespiratory fitness [9,10].

Emotional and social learning during childhood are important developmental processes through which children can learn and practice intra- and interpersonal skills [11]. School and outdoor activities are considered channels of social and emotional learning and therefore they play essential roles in the healthy development of children [12]. Most countries, in attempting to reduce the spread of COVID-19, have implemented social restrictions, lockdowns, and school closures, which, in turn, have negatively affected children’s educational performance, as well as their physical, mental, and social health [8].

Based on the above findings, and the lack of existing studies on the behavioral, social, and emotional impact of the COVID-19 pandemic on Saudi children, the present study aimed to clarify how the COVID-19 pandemic has affected Saudi children aged 3-15 years.

Our aim is to obtain research data that can inform the creation of post-COVID-19 public health programs that will promote and preserve healthy child development.

## Materials and methods

Objective

Primary Objective

To evaluate, using an assessment tool featuring closed-ended questions, the behavioral, emotional, and social changes experienced by Saudi children aged 3-15 years because of the COVID-19 pandemic.

Secondary Objective

To assess the ability of Saudi parents to manage the behavioral, emotional, and social changes of their children due to the COVID-19 pandemic.

Study design

This research was cross-sectional.

Study setting

This study was conducted in Saudi Arabia. As, at the time of the survey, measures to prevent the spread of COVID-19, including a ban on non-essential face-to-face meetings, were in effect, the survey was administered electronically. We contacted potential participants through mobile communication apps, such as Twitter, WhatsApp, and Facebook as the Saudi population commonly uses them.

Population

The target participants for this study were parents living in Saudi Arabia at the time of the survey and who had children between the ages of three and 15 years. Specifically, we applied the following inclusion and exclusion criteria:

Inclusion Criteria

Parents with Saudi nationality.

Having at least one child aged between 3-15 years.

Exclusion Criteria

Parents and families who live outside of Saudi Arabia.

Sample size and sampling

Based on the recent national survey [13], the estimated number of Saudi children from three to 15 is 5 million, however, 651 of the parents who met the inclusion and exclusion criteria were recruited for the present study, using a 5% margin of error and confidence level of 95%. Potential participants were identified through non-probability sampling and the electronic questionnaire was distributed to them. The questionnaire evaluated the recipients’ qualifications for inclusion in the research. Potential participants who did not meet the criteria were immediately excluded from the survey.

Operational definitions

Changes in Children’s Engagement in Health Behaviors

Behavior is an important determinant of health. From a psychological perspective, behaviors are humans’ external reactions to their surrounding environments, and all changes in behavior are triggered by positive and negative reinforcement factors originating from the environment or self-directed intentions [14]. In the present study, children’s level of engagement in health behaviors during the COVID-19 pandemic is compared with their engagement in such behaviors during the pre-pandemic period. Health behaviors were defined based on the Canadian 24-Hour Movement Guidelines for Children and Youth, which include the following recommendations for the level of physical activity, hours of sleep, and duration of sedentary behavior children should engage in within each 24-hour period [15]:

1) At least 60 min of moderate to vigorous physical activity, including different aerobic activities.

2) Several hours of light physical activity, including structured and unstructured light physical activities.

3) Sleep: consistent bed and wake-up times.

Children aged 5-13 years should receive 9-11 uninterrupted hours of sleep per night.

Those aged 14-17 years should receive 8-10 uninterrupted hours of sleep per night.

4) Sedentary behavior: No more than two hours of recreational screen time.

Children’s Social and Emotional Health

Childhood is a pivotal time for healthy development and the establishment of a foundation for future well-being. There are five core competencies for social and emotional learning: self-management, self-awareness, relationship skills, social awareness, and responsible decision-making [8,16]. Children’s social and emotional health are crucial influences on their current and future mental health and may affect their learning and development processes. Research indicates that children with social and emotional difficulties may have trouble when engaged in learning activities and show low self-esteem and poor school performance [16]. The present study aims to identify the social and emotional changes (if any) Saudi children have experienced because of the COVID-19 pandemic and subsequent social restrictions.

Questionnaire

Many of the items included in the questionnaire used for the present study were sourced, with permission from the authors, from a similar study on Arab Israeli children [17]. The behavioral assessment section of the present study’s questionnaire was based on the health-behavior recommendations stipulated in the Canadian 24-Hour Movement Guidelines mentioned above [15].

To ensure the face and content validity of the questionnaire, we translated its items from the original English into Arabic and then back translated into English. An expert panel of specialists in health quality, health informatics, community medicine, and mental health then revised the items. Reliability was tested by measuring internal consistency; Cronbach’s alpha coefficient was 0.72, indicating good reliability. Each section of the questionnaire is provided in Appendix A.

Study plan

This research was approved (NRJ21J/180/07) by the ethical and scientific committee of King Abdullah International Medical Research Center at King Abdul-Aziz Medical City, after which data collection was performed over a two-month period (July and August 2021). We scrupulously guarded the confidentiality of the participants’ data throughout the study. Prior to data collection, the participants provided their informed consent for study participation and publication of the data.

Data management and statistical analysis

The present study used SPSS statistical software package for Windows, version 20.0 (IBM Corp., Armonk, NY, USA) for data entry and statistical analysis. Quality control was performed during the coding and data-entry stages. In our analysis results, we present the data using descriptive statistics, with frequencies and percentages used for qualitative variables and means and standard deviations used for quantitative variables. Chi-square tests were used to measure the statistical significance of relationships between participants’ responses and various demographic characteristics. Finally, we compared children’s hours of sleep, engagement in physical activity, and screen time before and during the pandemic using paired McNemar’s tests.

## Results

Characteristics of study subjects

The sample comprised 651 participants in total; slightly over half (58%) were female. The mean age of the sample was 29±7 years (range: 20-60 years). Further, 94% of the participants were married (not divorced or widowed) and 72% of them had more than one child between the age of three and 15 years. In 63% of the families, both parents were employed (father and mother); in only 3.5% of the families were both parents unemployed (Table 1).

**Table 1:** Parents’ demographic characteristics (n=651) SD: standard deviation

Demographic characteristics	Frequency (n)	Percent (%)
Age		
Range (years)	20–60	
Mode (years), Mean ± SD (years)	22, 29 ± 7	
Gender of parent		
Male	271	41.6
Female	380	58.4
Marital status		
Married	614	94.3
Divorced	23	3.5
Widowed	14	2.2
No. of children aged 3–15 years		
One	179	28
Two	204	31
More than two	268	41
Employment status		
Both parents unemployed	23	3.5
Both parents employed	416	63.9
Only one parent employed	212	32.6

The respondents answered questions related to their children (Table 2). These items showed that the mean age of their children was 9±4 years, the majority of the children were living in apartments (63.9%), and that 89% of the children were living with both their father and mother.

**Table 2:** Children’s demographic characteristics (n=651) SD: standard deviation

Demographic characteristics	(n) Frequency	Percent (%)
Age		
Range (years)	3-15	
Mode (years), Mean ± SD (years)	4, 9 ± 4	
Gender of child		
Male	382	59
Female	269	41
Residence		
With father	6	0.9
With mother	62	9.5
With both parents	583	89.6
House type (accommodation)		
Lower-class house (traditional house)	23	3.5
Apartment (flat)	416	63.9
Villa	212	32.6

**Figure 1:**
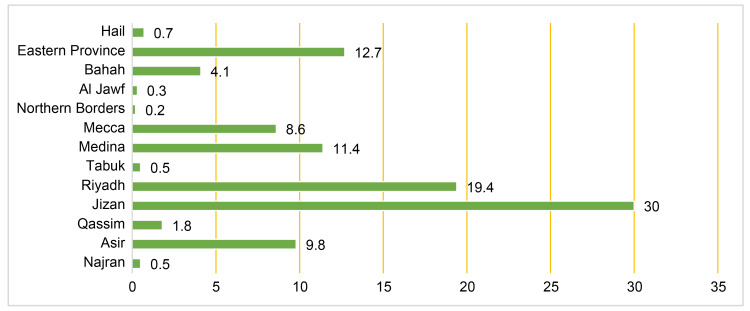
Percentage of participants from different Saudi Arabia’s 13 provinces (N=651)

**Figure 2:**
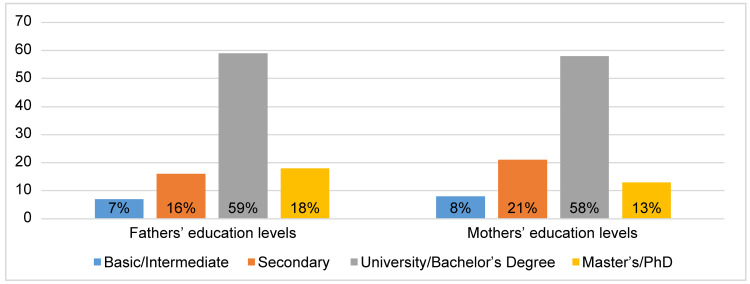
Percentage of parents among different education levels (N=651)

Figure 1 depicts the distribution of the participants across Saudi Arabia’s 13 provinces. This shows that 30% of the participants were from Jazan Province and 19% were from Riyadh. Figure 2 illustrates parents’ education levels, and shows that, for both the fathers and mothers, approximately 60% had at least a bachelor’s degree, indicating that the families had a generally high level of education.

Medical history

**Figure 3:**
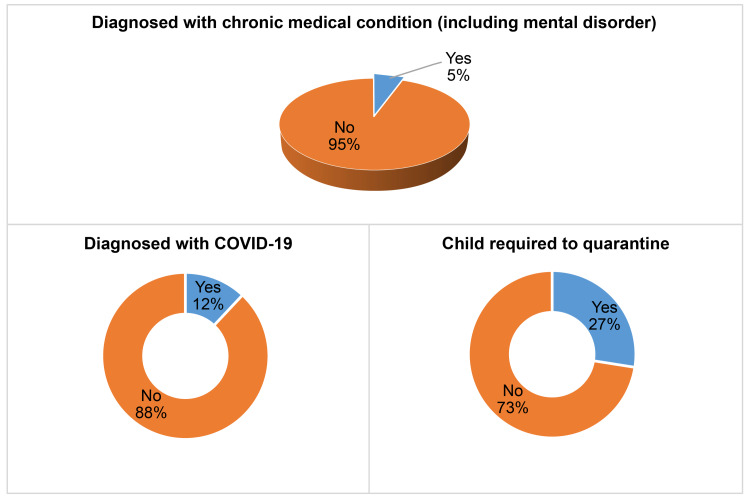
Children’s medical history (n=651)

Figure 3 illustrates the medical histories of the children and their parents. The majority of the children (95%) were healthy with no history of chronic medical conditions. Approximately 12% had recovered from COVID-19, and one-third had been required to quarantine due to COVID-19.

**Figure 4:**
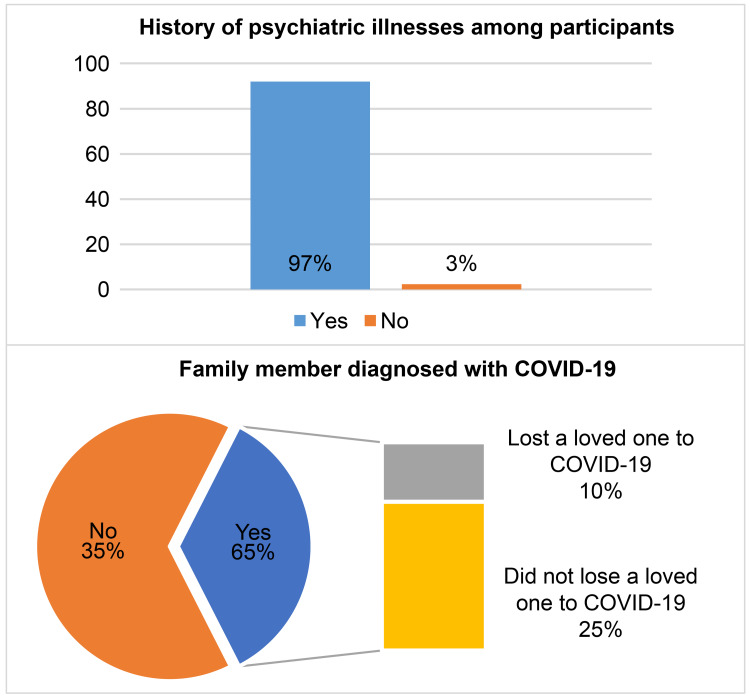
Participants’ medical history (N=651)

Concerning the parents’ medical history, only 3% of the respondents had previously been diagnosed with psychiatric disorders. At least one-third of the families had a relative who had been diagnosed with COVID-19, and 10% had lost a loved one because of COVID-19 (Figure 4).

Relationships between participants’ responses to the emotional assessment and their demographic characteristics and medical history

Table 3 shows that one-third of the children had asked to sleep in their parents’ bed during the outbreak period, and 10% had experienced a general deterioration in their vocabulary during the outbreak period. Moreover, almost one-third of the children demonstrated increased irritability and mood swings compared to the pre-pandemic period.

**Table 3:** Relationship between participants’ responses to the emotional assessment and their demographic characteristics (n=651) * Statistically significant at p<0.05

	Did your child ask more frequently to sleep in his/her parents’ bed during the outbreak period?			Did your child experience a general deterioration in his/her vocabulary across the outbreak period?					Did your child show increased irritability during the outbreak period?					Did you notice any mood swings in your child?				
	Yes (n)		No (n)	Yes (n)	No (n)				Yes (n)			No (n)		Yes (n)	No (n)			
Parents’ marital status																		
Married	179		435	50	564				187	427				188	426			
Divorced	5		18	3		20			9	14				8	15			
Widowed	2		12	1		13			2	12				2	12			
P value	0.36			0.69					0.28					0.37				
Education level																		
Basic/Intermediate	11		31	6				34	13	32				20	1			
Secondary	24		77	8				93	27	74				16	24			
University	118		270	30				358	115	273				24	77			
Master’s/PhD	33		84	10				107	43	74				107	281			
P value	0.58			0.55					0.55					0.003*				
Employment status																		
Both parents un-employed	4		19	3		20			5	18				7	16			
Both parents employed	69		143	15		197			55	157				61				151
Only one parent employed	113		303	36			380		138	278				130				286
P value	0.178			0.58					0.166					0.81				
House type (accommodation)																		
Lower-class house (traditional house)	11		14	2			23		8				17	12		13		
Apartment (flat)	110		233	31			312		110				233	124		219		
Villa	65		218	21				262	80				203	62		221		
P value	0.009*			0.76					0.58					0.001*				
Residence of child																		
With father	0		6	1				5	1			5		1		5		
With mother	6		56	21				41	19			43		21		41		
With both parents	48		535	176				407	178				405	176			407	
P value	0.7			0.63					0.76					0.63				
No. of children aged 3–15 years																		
One child	51	128		24			155		42			137		49	130			
Two children	67	137		12			192		76			128		75	129			
More than two children	68	200		18			250		80		188			74	194			
P value	0.20			0.014*					0.013*					0.05				
Gender of child																		
Male	273	107		25			355		102		278			102	278			
Female	192	79		29			242		96		175			96	175			
P value	0.78			0.06					0.019*					0.019*				
Age of child	Mean ( 7 )	Mean (10)		Mean ( 7 )			Mean (9)		Mean ( 9 )		Mean (8)			Mean ( 8.9 )	Mean (8.7)			
P value	0.001*			0.007*					0.73					0.8				

Table 3 displays the non-significant level of association between participants’ responses to the emotional assessment and the following factors: marital status; employment status; and whether the children were living with their mother, father, or both.

However, data analysis showed that most children had low levels of emotional disturbance, and that “no” responses to emotional assessment questions were more likely if the parents had a high level of education and the children were living in an apartment or villa and had more than two siblings. Related to gender, male children were more likely to show emotional stability compared to female children, with statistically significant differences in the areas of irritability and mood swings (p<0.05). On the other hand, younger children were significantly more likely to ask to sleep in their parents’ bed and to show vocabulary deterioration (p<0.05).

Table 4 displays the associations between participants’ responses to the emotional assessment and their medical history. This shows that children who had a chronic medical condition, had been diagnosed with COVID-19, or had been required to quarantine as a result of COVID-19 were significantly more likely to experience high emotional disturbance (p<0.05). In addition, children who had family members who had contracted COVID-19 or who had lost a loved one due to COVID-19 infection were also more likely to report emotional disturbance.

**Table 4:** Relationship between participants’ responses to the emotional assessment and medical history (n=651) * Statistically significant at p<0.05

	Did your child ask to sleep in his/her parents’ bed during the outbreak period?		Did your child experience a general deterioration in his/her vocabulary across the outbreak period?					Did your child show increased irritability during the outbreak period?				Did you notice any novel mood swings in your child?					
	Yes (n)	No (n)	Yes (n)	No (n)				Yes (n)		No (n)		Yes (n)	No (n)				
History of diagnosis of chronic medical conditions (among children)																	
No (n)	15	21	8			28		16	20			18				18	
Yes (n)	171	444	46			569		182	433			180				435	
P value	0.074		0.002*					0.06				0.009*					
History of COVID-19 diagnosis (among children)																	
No (n)	22	54	4	72				31	45			31					45
Yes (n)	164	411	50		525			167	408			167					408
P value	0.93		0.3					0.03*				0.03*					
History of being required to quarantine as a result of COVID-19 (among children)																	
No (n)	67	112	17				162	76		103		67		112			
Yes (n)	119	353	37				435	122			350	131			341		
P value	0.002*		0.49					0.001*				0.017*					
Family member diagnosed with COVID-19																	
No (n)	73	155	25				203	82	146			76	152				
Yes (n)	113	310	29				394	116	307			122	301				
P value	0.15		0.07					0.02*				0.23					
History of losing a friend or family member to COVID-19																	
No (n)	51	133	17			167		69			115	66		118			
Yes (n)	135	332	37				430	129			338	132		335			
P value	0.76		0.58					0.01*				0.058					

Relationships between participants’ responses to the social assessment and their demographic characteristics and medical history

Table 5 shows that 22% of the participants reported that their children appeared calmer during the pandemic period, and 14% felt that their children were more thoughtful during this period. They described the presence of guests in the home during the pandemic as unpleasant for almost one-third of the children. They also reported that two-thirds of children showed good adherence to precautionary measures (wearing a mask, distancing, using hand sanitizer).

**Table 5:** Relationship between participants’ responses to the social assessment and their demographic characteristics (n=651) * Statistically significant at p<0.05 ANOVA: analysis of variance

	During the COVID-19 pandemic, did your child appear quiet and clam?			Did your child appear more reasonable or thoughtful during the COVID-19 pandemic?					Does the presence of guests in the house cause your child to feel discomfort because of a fear of COVID-19 infection?					How do you evaluate your child's application of preventive measures (wearing a mask - distancing - using hand sanitizer)?			
	Yes (n)		No (n)	Yes (n)	No (n)				Yes (n)			No (n)		Bad(n)	Poor (n)	Good (n)	Excellent (n)
Parents’ marital status																	
Married	137		477	89	525				169	445				17	49	417	131
Divorced	6		17	4		19			5	18				0	1	13	9
Widowed	3		11	3		11			5	9				1	1	6	6
P value	0.9			0.7					0.65					0.15			
Education level																	
Basic/Intermediate	13		32	11				34	14	31				2	3	30	10
Secondary	16		85	13				88	30	71				2	10	62	27
University	80		308	55				333	102	286				12	30	264	82
Master’s/PhD	37		80	17				100	33	84				2	8	80	27
P value	0.03*			0.42					0.8					0.38			
Employment status																	
Both parents unemployed	2		21	3		20			6	17				1	5	15	2
Both parents employed	38		174	30		182			58	154				4	14	157	37
Only one parent employed	106		310	63			353		115	301				13	32	264	107
P value	0.02*			0.9					0.9					0.015*			
House type (accommodation)																	
Lower-class house (traditional house)	6		19	3			22		6				19	3	1	12	9
Apartment (flat)	83		260	53			290		90				253	9	36	229	69
Villa	57		226	40				243	83				200	6	14	195	68
P value	0.47			0.83					0.63					0.003*			
Residence of child																	
With father	1		5	0				6	0			6		0	0	6	0
With mother	17		45	22				40	24			38		2	5	37	18
With both parents	128		455	74				509	155				428	16	46	393	128
P value	0.58			0.001*					0.04*					0.5			
No. of children aged 3–15 years																	
One child	42	137		29			150		44			135		6	19	114	40
Two children	42	162		23			181		47			157		4	20	136	44
More than two children	62	206		44			224		88		180			8	12	186	62
P value	0.7			0.24					0.03*					0.23			
Gender of child																	
Male	85	295		56			324		102		278			10	32	255	83
Female	61	210		40			231		77		194			8	19	181	63
P value	0.96			0.9					0.68					0.8			
Age of child	Mean ( 7 )	Mean (9)		Mean ( 8 )			Mean (11)		Mean ( 8 )		Mean (10)			ANOVA test F=16			
P value	0.001*			0.001*					0.01*					0.001*			

Regarding the relationship between participants’ responses to the social assessment and their demographic characteristics, Table 5 shows that the majority of the children had relatively low adaption to the pandemic. Children who had married and highly educated parents, at least two siblings, and who were living with both parents in a villa or apartment were more likely to appear calmer and more thoughtful. Regarding employment status, a statistically significant relationship was observed between families with only one employed parent and children who were not calm (p<0.05). Although there were no significant differences between the male and female children regarding the social assessment questions, older children showed more adaptive behavior compared to younger children at a statistically significant (p<0.05) level.

Regarding the association between the children’s medical history and the social assessment responses, Table 6 shows that children diagnosed with a chronic medical condition, diagnosed with COVID-19, or required to quarantine due to COVID-19 showed less adaptive behavior. Moreover, participants reported a high level of social disturbance among children who had a family member who had contracted COVID-19 or who had lost a loved one because of COVID-19.

**Table 6:** Relationship between participants’ responses to the social assessment and medical history (n=651) * Statistically significant at p<0.05

	During the COVID-19 pandemic, did your child appear quiet and clam?		Did your child appear more reasonable or thoughtful during the COVID-19 pandemic?					Does the presence of guests in the house cause your child to feel discomfort because of fear of COVID-19 infection?				How do you evaluate your child's application of preventive measures (wearing a mask - distancing - using hand sanitizer)?			
	Yes (n)	No (n)	Yes (n)	No (n)				Yes (n)		No (n)		Bad (n)	Poor (n)	Good (n)	Excellent (n)
History of diagnosis of chronic medical conditions (among children)															
Yes (n)	17	19	8		28			11	25			0	1	26	9
No (n)	129	486	88		527			168	447			18	50	410	137
P value	0.001*		0.192					0.67				0.436			
History of COVID-19 diagnosis (among children)															
Yes (n)	19	57	16				60	25	51			5	2	53	16
No (n)	127	448	80				495	154	421			13	49	383	130
P value	0.56		0.09					0.26				0.053			
History of being required to quarantine as a result of COVID-19 (among children)															
Yes (n)	45	134	28		151			47	132			9	9	119	42
No (n)	101	371	68			404		132	340			9	42	317	104
P value	0.37		0.69					0.63				0.06			
Family member diagnosed with COVID-19															
Yes (n)	51	177	36			192		65			163	12	16	146	54
No (n)	95	328	60				363	114			309	6	35	290	92
P value	0.97		0.58					0.67				0.03*			
History of losing a friend or family member to COVID-19															
Yes (n)	47	137	37				147	65		119		9	16	114	45
No (n)	99	368	59				408	114			353	9	35	322	101
P value	0.23		0.01*					0.005*				0.11			

Behavioral changes during the pandemic

Table 7 shows that most parents observed negative behavioral changes in their children comparing the pandemic to the pre-pandemic period, with the children spending fewer hours engaging in physical activities and sleeping, and more time viewing screens. Moreover, although there was no statistically significant change in children’s sleeping hours before and during the pandemic, significant (p<0.05) changes between these points were found for time spent performing physical activities and time spent viewing screens.

**Table 7:** Differences in children’s behaviors between the period before the COVID-19 pandemic and during the pandemic * Statistically significant at p<0.05

	Before n (%)	After n (%)	McNemar’s test	P value
Hours of sleep			0.12	0.72
Less than nine hours	209 (32)	214 (33)		
More than nine	442 (68)	437 (67)		
Use of screens			136	0.001*
Two hours or less	239 (37)	83 (13)		
More than two hours	412 (63)	568 (87)		
Physical activity			88	0.001*
Less than one hour	189 (29)	321 (49)		
One hour or more	462 (71)	330 (51)		

Most participants reported trying to mitigate the negative impact of the pandemic on their children. Specifically, 85.9% of parents tried to find suitable indoor leisure activities for their children. Despite the enforcement of strict preventive measures, two-thirds of parents tried to find outdoor recreational activities for their children. Only half of the parents, however, limited screen use, including TV, smartphones, and tablets, to a maximum of two hours. On the other hand, communication with children was reported by 89% of parents, and this may be associated with the finding that 92% of the parents did not seek help from a child mental-health professional (Table 8).

**Table 8:** Coping actions initiated by parents

	(n) Frequency	Percent (%)
Have you tried to find an indoor leisure activity or hobby that your child can enjoy?		
Yes	559	85.9
No	91	14.1
Have you tried to find an outdoor recreational activity or hobby that your child can enjoy while adhering to preventive measures?		
Yes	453	69.6
No	198	30.4
Have you tried to enhance communication with your child by engaging in discussions and performing active listening?		
Yes	581	89.2
No	70	10.8
Have you begun to limit your child’s hours of screen use, including TV, smartphones, and tablets, to a maximum of two hours?		
Yes	384	59
No	267	41
Have you sought help from a child mental-health professional?		
Yes	46	7.1
No	605	92.9

## Discussion

The COVID-19 pandemic has influenced the lifestyles of children and families around the world, with many studies reporting that the pandemic has had multiple notable effects on children’s psychological and physical health [11,12]. Among parents who responded to the questionnaire distributed in the present study, almost one-third reported that their children had asked to sleep in their parents’ beds, and 30% said that their children had become more irritable and had developed mood swings. Overall, young children, girls, and children with low socioeconomic status (determined through considering parents’ level of education, type of house lived in, and parents’ employment status) tended to show higher levels of emotional disturbance during the COVID-19 pandemic.

The present findings concerning children’s desire to avoid sleeping alone and their expressions of fears and anxiety accord with the results of recent studies on the COVID-19 pandemic’s impact on psychological health [7,12,17]. The present study’s findings regarding the negative effects of the pandemic on children in families with low socioeconomic status are consistent with those reported in studies conducted in the United States [18] and in Canada [19]. Both studies found that, during the COVID-19 pandemic, low-income families experienced more stress and poorer health outcomes compared to high-income families.

 Although social restriction is an important prophylactic measure during pandemics, it is associated with numerous negative effects, such as stress and anxiety [20,21]. The present study assessed the adaptation behaviors of children to COVID-19-related social restrictions. The results showed that less than one-third of the responding parents felt that their children appeared calmer and more thoughtful during the pandemic. Moreover, almost one-third of the parents reported that their children were afraid to have guests in their homes, thinking that these visitors could infect them. Furthermore, studies [20,21] among school-age children have shown that school closures might increase loneliness and decrease children’s connection with their friends, which, in turn, correlates with mental-health effects, such as anxiety and depression.

In addition to social restrictions, the public should follow other measures for preventing COVID-19 infection, such as wearing masks, regularly practicing hand hygiene and, recently, getting the recommended COVID-19 vaccine. Public adherence to these precautions is crucial for preventing the spread of the disease. Two-thirds of the parents surveyed in the present study reported that their children were committed to adhering to preventive measures. This result is consistent with that of a previous Saudi-Arabia-based study [22] that assessed Saudi parents’ compliance with COVID-19 precautions relating to children; the study revealed that Saudi parents showed a high level of adherence to and compliance with preventive measures for their children.

Because of lockdowns and school closures, family members spent more time together during the pandemic. Sibling relationships are an essential part of children’s lives, and have positive effects on children’s development; preservation of sibling relationships is important for coping with potential stressors [23]. In Saudi culture, such relationships are greatly valued and are encouraged by the Islamic religion. Previous researchers have observed a link between poor sibling relationships and adverse family circumstances [24,25]; this association could be attributed to parents’ relationship, family members’ well-being, and social structure. In accordance with these previous findings, in the present study we observed that, during the pandemic, families with more than two siblings were more adaptive and had lower levels of emotional disturbance.

A paradoxical finding of the present study is that younger children experienced greater social and emotional disturbance during the pandemic compared to older children. Older children’s higher levels of awareness and their ability to recognize various different dimensions of the current situation may account for this difference. This finding contrasts with those of a previous study [25] examining Arab Israeli children which found that pre-kindergarteners and young children are better protected from emotional and social disturbance compared to older children, suggesting that young children are unaware of the reality of the disease.

Many research studies have examined the psychological consequences of COVID-19 since the beginning of the outbreak [7,26]. A previous study found that children who had been required to quarantine showed levels of post-traumatic stress that were four times higher than those of children who did not quarantine [27]. Similarly, a Saudi-Arabia-based study reported that one-third of the children in its sample experienced mild levels of post-traumatic stress due to COVID-19-related quarantine/lockdown measures [28]. In congruence with these findings, the present study found that children not diagnosed with COVID-19 and/or not required to quarantine appeared calmer, more thoughtful, and more emotionally stable.

The present study examined changes in the children’s engagement in physical activity, leisure screen time, and sleep time as a result of the COVID-19 pandemic, using the Canadian 24-Hour Movement Guidelines for Children and Youth to define healthy levels of these behaviors. The results showed that, during the pandemic, children were less active and engaged in more recreational screen-based activities. Other studies have reported similar negative changes [9,17]. These changes may result from the psychological consequences of social restrictions. Notably, a recent systematic review of research from Arab-speaking countries linked changes in such behaviors with higher levels of adiposity, behavioral problems, depression, and low self-esteem [29].

The current study set a secondary objective of assessing Saudi parents’ strategies for addressing behavioral, emotional, and social changes in their children. The findings showed that the majority of the parents made significant efforts to improve their children’s behaviors by encouraging engagement in outdoor/indoor recreational activities and limiting the number of hours of screen time. A previous Canadian study found that engagement in such recreational activities helps to mitigate the mental-health challenges exacerbated by social restrictions [30].

The present study has some limitations. Our survey had a cross-sectional design, useful for formulating hypotheses about associations, but unable to detect cause-effect relationships. The data-gathering method was self-report, which can be subject to recall bias. Finally, the current pandemic situation hampered our efforts to procure a larger sample. Such a sample would have increased the power of the study and enhanced the generalizability of the research findings. Despite these limitations, however, our study provides key insights into the behavioral, emotional, and social changes experienced by Saudi children during the COVID-19 pandemic.

## Conclusions

The emotional, social, and behavioral reactions of children to the COVID-19 pandemic represent public-health challenges. Children are more vulnerable to its negative psychological effects. The psychological impact of the pandemic on some children has been exacerbated by the sudden social isolation from friends and family experienced due to social restrictions, which have forced them to spend most of their time at home, engaging in increased screen time and less physical activity. Indeed, the current pandemic has dramatically demonstrated the essential need for purposeful intervention programs to support parents in promoting the health of their children (mental, emotional, and social) during crises in accordance with the social, cultural, and religious characteristics of families in Saudi Arabia.

## Appendices

Appendix A

The questionnaire used in this study was electronic, self-administered, and comprised of six sections, as described below.

First Section

This section concerned demographic data, and measured the following characteristics:

Parents’ characteristics: Age, gender, education level, nationality, marital status, number of children aged 3-15 years, employment status, current profession, and working hours.

Children’s characteristics: Age, gender, house type (e.g., apartment, villa), and residency status (i.e., living with mother or father or both).

Second Section

This section concerned medical histories of the parents and children, and featured the following items:

Parents’ medical history:

“Have you ever been diagnosed with a mental or psychological disorder?”

“Have you been diagnosed with COVID-19?”

“Have you been requested to quarantine at home?”

“Other than yourself, have any of your family members been diagnosed with COVID-19?”

“Have you lost any family members to COVID-19?”

Children’s medical history:

“Has your child ever been diagnosed with a chronic medical condition?”

“Has your child ever been diagnosed with a mental, psychological, or behavioral disorder?”

“Has your child been diagnosed with COVID-19?”

“Has your child been asked to quarantine at home?”

“If your child has lost any close friends or family members to COVID-19, does your child know that the death was due to COVID-19?”

Third Section

This section was an assessment of child emotional health, and featured the following closed-ended questions:

“Did your child ask to sleep in his/her parents’ bed during the outbreak period?”

“Did your child experience a general deterioration in his/her vocabulary across the outbreak period?”

“Did your child show increased irritability during the outbreak period?”

“Did you notice any novel mood swings in your child?”

Fourth Section

This section assessed the children’s social functioning, and featured the following closed-ended questions:

“During the COVID-19 pandemic, did your child appear quiet and clam?”

“Did your child seem more reasonable or thoughtful during the COVID-19 pandemic?”

“Did the presence of guests in the house cause your child to feel discomfort as a result of a fear of COVID-19 infection?”

“Did your child avoid leaving the house because of a fear of COVID-19 infection?”

“How do you evaluate your child’s application of preventive measures (wearing a mask, performing social distancing, using hand sanitizer)?”

Fifth Section

This section was a child behavioral assessment, and featured the following closed-ended questions:

“How many hours per day did your child sleep during the pandemic, and how many hours per day did he/she sleep before the pandemic?”

“How many hours per day did your child spend viewing digital screens, including TV, tablets, and phones, during the pandemic, and how many hours per day did he/she spend viewing screens before the pandemic?”

“How many hours per day did your child spend performing physical activity during the pandemic, and how many hours per day did he/she spend performing such activities before the pandemic?”

Sixth Section

This section concerned parents’ use of adaptive and coping strategies and recommended interventions, as measured through the lens of a socio-ecological model (intra- and inter-individual) [26]. The following items were included:

“Have you tried to find an indoor leisure activity or hobby that your child can enjoy?”

“Have you tried to find an outdoor recreational activity or hobby that your child can enjoy while adhering to preventive measures?”

“Have you tried to enhance communication with your child by engaging in discussions and performing active listening?”

“Have you tried to limit your child’s screen use, including TV, smartphones, and tablets, to a maximum of two hours?”

“Have you sought help from a child mental health professional?”
